# HCViT‐Net: Hybrid CNN and multi scale query transformer network for dermatological image segmentation

**DOI:** 10.1002/acm2.70385

**Published:** 2025-11-27

**Authors:** Wei Jiao, Jianghui Xu, Yijiao Fang, Jiaojiao Huang, Yujie Zhu, Dandan Ling

**Affiliations:** ^1^ Department of Anesthesiology Fudan University Shanghai Cancer Center Shanghai China; ^2^ Department of Dermatology Shanghai Ninth People's Hospital Shanghai China; ^3^ School of Medicine Shanghai Jiaotong University Shanghai China

**Keywords:** boundary refinement, medical image segmentation, multi‐scale features, skin lesion

## Abstract

**Background:**

Dermoscopic lesion segmentation is crucial for dermatology, yet existing methods struggle to integrate global context with local details under the efficiency constraints required for clinical use.

**Purpose:**

We aim to develop a lightweight model that simultaneously captures long‐range spatial dependencies and preserves fine‐grained boundary details for dermoscopic lesions. The method is designed to achieve a favorable accuracy–efficiency trade‐off, thereby improving segmentation performance and ensuring potential for practical clinical deployment.

**Methods:**

Proposing a lightweight hybrid model, HCViT‐Net, featuring an encoder–decoder architecture. It incorporates a multi‐scale query transformer (MSQFormer) into each stage of its convolutional encoder to efficiently capture global, multi‐scale context. Furthermore, a wavelet‐guided attention refinement module (WARM) is introduced on the highest‐resolution skip connection to selectively enhance high‐frequency boundary details and bridge the semantic gap between the encoder and decoder, thus improving model performance.

**Results:**

Evaluated on ISIC 2017 and 2018, our model achieved mean intersection‐over‐union (mIoU) of 87.76% and 87.45%, respectively. With only 5.76M parameters and 7.51 GFLOPs, it demonstrates performance competitive with existing methods at a significantly lower computational cost.

**Conclusions:**

HCViT‐Net achieves an excellent accuracy–efficiency trade‐off. It improves segmentation accuracy with a low computational footprint, showing strong potential for practical deployment in dermatology workflows.

## 1 INTRODUCTION

Melanoma, characterized by aggressive invasiveness, high metastatic potential, and ele vated mortality, has become one of the fastest‐growing malignancies worldwide.^1^ In clinical settings, dermatologists must manually identify and delineate lesions via dermatoscopic imaging—a diagnostic procedure critically dependent on clinician experience and technical proficiency. Substantial evidence demonstrates that automated lesion segmentation in skin imaging enhances the accuracy of abnormality detection by both clinicians and AI diagnostic systems, thereby providing an objective foundation for early screening and differential diagnosis.^2^, ^3^

The rapid evolution of deep learning and computer vision^4^ has revolutionized medical image segmentation, yielding unprecedented gains in accuracy. The fully convolutional network (FCN)^5^ pioneered pixel‐level CNN segmentation, and subsequent models—for example, DeepLab^6^ with atrous spatial pyramid pooling (ASPP) for larger receptive fields, and U‐Net^7^ with encoder–decoder skip connections—have refined multi‐scale context capture and fine‐detail recovery.

**FIGURE 1:**
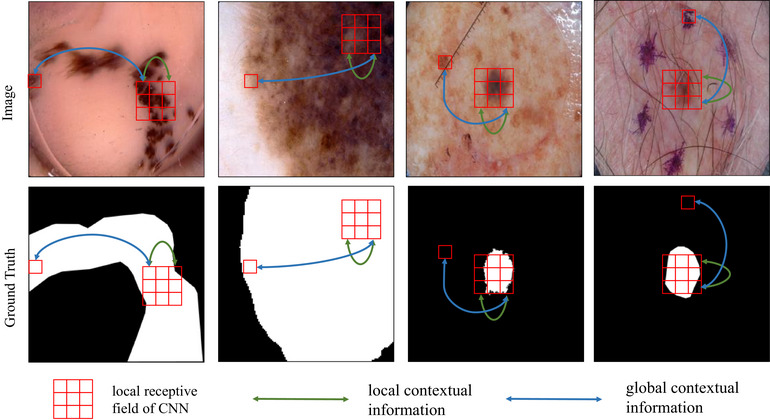
Illustration of the global (blue curves) and local (green curves) contextual information in dermatological images. By establishing global feature dependencies across image regions, the model can capture the global morphological characteristics of lesions and enhance its discriminative power against lesion‐mimicking artifacts, thereby improving segmentation precision.

While these CNNs‐based segmentation models^5^, ^6^, ^7^ have achieved remarkable success, the inherently local receptive fields of convolutional layers limit global dependency modeling, a key drawback in skin lesion segmentation. As illustrated in the first two columns of Figure 1, skin lesions in dermoscopic images often exhibit significant spatial occupancy that may exceed the local receptive fields of CNNs. The intrinsic locality of convolution operations fundamentally limits the model's capacity to capture global morphological characteristics of lesions. Furthermore, the latter two columns of Figure 1 demonstrate that skin lesions can present textural similarities to benign skin features. Establishing global feature dependencies across image regions proves crucial for enhancing the model's discriminative power against lesion‐mimicking artifacts, thereby improving segmentation precision.

Therefore, due to the superior performance of ViTs^8^, ^9^ architectures in establishing long‐range dependencies, some works^10^, ^11^, ^12^ have introduced them into segmentation models to fully extract global information from images. For example, SwinUNet^10^ employs Swin Transformer^9^ as the backbone network to construct a U‐shaped medical image segmentation network composed entirely of ViT.

These pure‐ViT‐based segmentation methods can fully extract global information from images. However, limited by the computational mechanism of self‐attention, ViTs typically incur a quadratic computational cost in terms of token quantity, while also lacking the ability to preserve local details during feature extraction. Thus, some works combining CNN and ViT within a single model to overcome this issue. For instance, TransUNet^12^ and TransFuse^13^ integrates ViT‐R50^8^ as the backbone network for feature extraction, enabling the network to simultaneously capture global and local features. Nevertheless, constrained by the high computational cost of ViTs, these methods can only incorporate ViT modules in regions with smaller feature maps. Studies in non‐local^14^ have shown that integrating the global information module into the model's shallow layers—where feature maps typically have larger spatial dimensions—yields greater performance gains. Moreover, inserting global feature modeling modules at multiple stages can progressively yield better results. In addition, due to the fixed window size and token dimension, existing advanced vision transformer modules^8^, ^9^ often lack internal multi‐scale information, which is critical for the accuracy of segmentation results.^15^, ^16^

**FIGURE 2:**
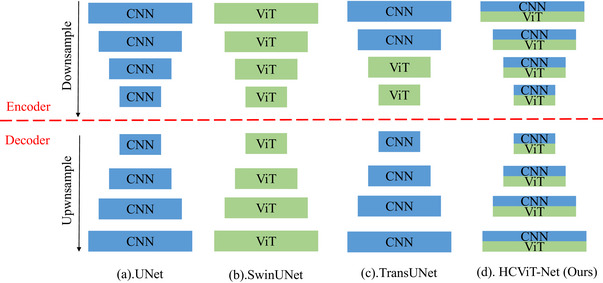
Illustration of the differences in model architecture between previous methods and ours. We combine CNN and ViT at all stages of the model to fully exploit the advantages of these two computing paradigms. ViT, vision transformer.

To address above problems of existing ViT modules,^8^, ^9^, ^12^ we propose a novel architecture named HCViT‐Net that systematically integrates both CNN and ViT components across all stages of the model. Figure 2 illustrates the architectural distinctions between our proposed method and existing pure CNN‐based, pure ViT‐based, and hybrid CNN‐ViT models. Our method uniquely enables comprehensive learning of both local and global features at every stage of the model, addressing limitations of prior works that often prioritize one type of information over the other. We design a lightweight self‐attention approach featuring multi‐scale key‐value (K‐V) reduction. This solution not only significantly reduces the computational overhead of standard self‐attention (maintaining our HCViT‐Net's advantage in model complexity), but also constructs intrinsic multi‐scale representations within ViT, thereby compensating for the absence of internal multi‐scale information in competitive ViT architectures.

Additionally, in the skip connections of UNet^7^ architecture, the shallow features generated by the first encoder layer preserve rich spatial details but lack sufficient semantic representation, while the corresponding deep decoder features contain abundant semantic information at the cost of spatial detail loss due to repeated downsampling. This inherent semantic gap leads to significant performance degradation when using conventional fusion methods direct summation or channel concatenation. To address this issue, we propose a feature refinement module named wavelet‐attention refinement module (WARM), which can adaptively establishes cross‐level feature correlations, effectively bridging the semantic gap and consequently improving segmentation accuracy.

Consequently, the contributions in this work can be summarized as follows:

1. To combine the local detail extraction capabilities of CNNs with the global context modeling strengths of ViTs, we introduce HCViT‐Net, a novel hybrid CNN–ViT architecture that integrates both CNNs and ViTs at all stages of the model—in contrast to existing approaches which typically insert ViT modules only in a few stages—thereby enabling the full extraction of local and global contextual information.
2. To address the high computational cost and the lack of internal multi‐scale information in existing ViT modules, we propose the multi‐scale query transformer (MSQFormer), which compresses key and value tensors at multiple resolutions, thereby enabling global and multi‐scale self‐attention with significantly reduced computational complexity. This characteristic is pivotal in maintaining the low computational overhead of our proposed HCViT‐Net.
3. To reduce the semantic gap between early encoder features and late decoder features, we propose the wavelet‐attention refinement module (WARM), which based on wavelet transform attention. WARM decomposes the encoder's feature maps using wavelet transform and then leverages high‐level semantic information from the decoder to guide the extraction of effective low‐level features, thereby improving the final segmentation accuracy.
4. We conduct extensive evaluations on ISIC 2017 and ISIC 2018 benchmarks, demonstrating that HCViT‐Net consistently outperforms pure CNNs, pure ViTs, and existing hybrid methods in segmentation accuracy while maintaining competitive model size and computational cost. This dual achievement underscores the model's significant potential for seamless integration into clinical workflows, offering a powerful tool to assist dermatologists in performing more timely and precise diagnostic assessments.

## 2 RELATED WORKS

### 2.1 Pure CNN‐based segmentation methods

Since the advent of fully convolutional networks (FCN),^5^ end‐to‐end convolutional models have become the cornerstone of modern semantic segmentation. The DeepLab^6^ family of models enhances contextual modeling by introducing atrous (dilated) convolutions, which enlarge receptive fields without downsampling, and by employing atrous spatial pyramid pooling (ASPP) for multi‐scale context aggregation. In the medical imaging domain, the U‐Net^7^, ^17^ architecture addresses challenges of limited data and the need for precise localization through a symmetric encoder–decoder structure with skip connections. This design directly shuttles encoder features to the decoder, preserving spatial details that might be lost during downsampling while enabling hierarchical feature extraction.

These CNN‐based segmentation models^5^, ^6^, ^7^, ^17^ have achieved remarkable success. However, limited by their local receptive fields, standard convolutional operations struggle to model long‐range dependencies.

### 2.2 Pure ViT based segmentation methods

The advent of the vision transformer (ViT)^8^ has spurred researchers to explore extending its powerful global modeling capabilities to dense prediction tasks. ViT's core concept involves dividing an image into a sequence of non‐overlapping patches, successfully adapting the standard Transformer^8^ architecture for image classification. In the realm of semantic segmentation, early applications of ViT often treated it as a pixel‐wise encoder. For instance, the SETR^18^ model utilizes a ViT encoder followed by upsampling and refinement stages to recover high‐resolution masks. Similarly, in medical image segmentation, Swin‐UNet^10^ adeptly employs the Swin Transformer^9^ to construct a U‐shaped segmentation network, achieving leading segmentation results.

These pure ViT‐based methods can effectively excavate global dependencies in images, but are constrained by the limitations of existing ViT modules. They fail to preserve adequate local details during feature extraction,^19^ and additionally lack multi‐scale query capability within their internal modules.

### 2.3 Hybrid CNN and ViT segmentation methods

In medical image segmentation, hybrid CNN–ViT architectures have emerged to leverage the local feature extraction of convolutions alongside the global context modeling of self‐attention. TransUNet^12^ pioneered this line by embedding a ViT module in the bottleneck of a U‐shaped CNN, allowing long‐range dependencies to guide upsampling and yielding strong performance on abdominal and cardiac CT tasks. Building on this idea, CoTr^20^ introduced a deformable self‐attention mechanism that sparsely attends to salient medical features, reducing both memory footprint and computational overhead while preserving fine‐grained structures.

These methods integrate CNNs and ViTs within one model, but due to the high computational cost of self‐attention mechanisms, these ViT modules can typically only be inserted at the deeper stages of the model. This inherently limits the model's ability to perform global information modeling at other stages and consequently restricts further improvements in segmentation accuracy.

## 3 METHODS

### 3.1 Overview

**FIGURE 3:**
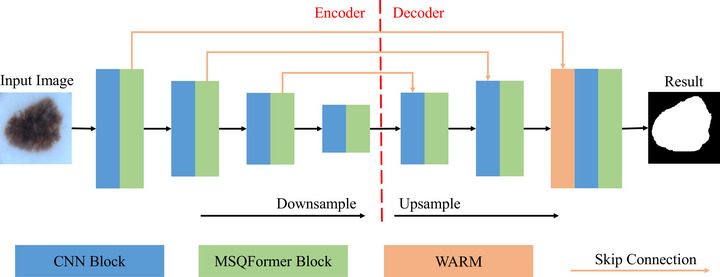
The architecture of the proposed HCViT‐Net.

Figure 3 illustrates the detailed architecture of our proposed model. The input image is progressively downsampled by the encoder and then restored to its original resolution by the decoder. Our baseline is a U‐shaped network composed solely of CNN blocks. To enrich each stage with global context, we insert the proposed multi‐scale query transformer (MSQFormer) block immediately after every CNN block. Multiple skip connections are utilized to pass low‐level details from the encoder to the decoder. For the two smaller‐sized skip connections, features are fused with the upsampled decoder output via element‐wise addition. Notably, the largest feature map from the earliest encoder stage contains minimal semantic content and significant noise. We first refine these features with our proposed wavelet‐attention refinement module (WARM), and then fuse them with the upsampled decoder output using the same additive method.

### 3.2 CNN block

**FIGURE 4:**
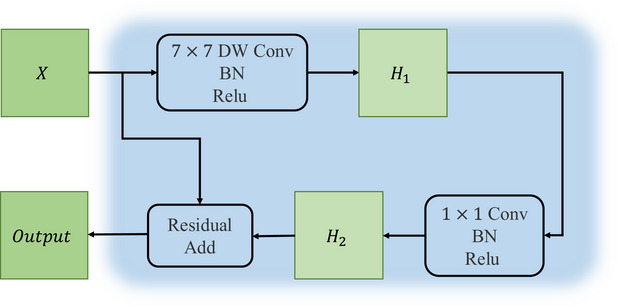
The architecture of the CNN Block in HCViT‐Net.

Driven by considerations for computational complexity and parameter efficiency, we built our custom CNN block drawing inspiration from the design philosophy of the classic lightweight network, MobileNet.^21^ As shown in Figure 4, this module consists of a 7×7 depthwise convolution (DWConv) layer followed by a 1×1 standard convolution layer. The 7×7 depthwise convolution captures spatial contextual information across a wide receptive field at a very low computational cost, while the 1×1 standard convolution performs information interaction and recombination across the channel dimension to generate new, more expressive feature representations. Furthermore, each convolution layer is succeeded by a batch normalization (BN) layer and a rectified linear unit (ReLU) activation function.

Let the input feature map be $X\in R^{B\times C\times H\times W}$, where B is the batch size, C is the channel dimension, H is the height and W is the width, the above steps can be represented by formulas as:

(1) $$H_{1}=\text{ReLU}\left(\text{BN}\left({\text{DWConv2D}}_{k=7,s=1}\left(X\right)\right)\right)\in R^{B\times C\times H\times W}.$$

(2) $$H_{2}=\text{ReLU}\left(\text{BN}\left({\text{Conv2D}}_{k=1,s=1}\left(H_{1}\right)\right)\right)\in R^{B\times C\times H\times W}.$$

(3) $$Z=X+H_{2}\in R^{B\times C\times H\times W}$$

where ReLU is the rectified linear unit activation function and BN is the batch norm.

### 3.3 Multi‐scale query transformer block

To effectively segment dermatological lesions, which inherently exhibit significant variations in size and shape, the model must be capable of processing contextual information from multiple resolutions simultaneously.^15^, ^16^ Therefore, the motivation for our multi‐scale key‐value compression strategy is twofold: on one hand, it drastically reduces the computational overhead of the self‐attention^8^ mechanism, making its deployment across all network stages feasible. On the other hand, and more importantly, it empowers a single query vector to simultaneously interact with contextual information aggregated from different spatial scales. This capability is clinically crucial, as it allows the model to be equally adept at capturing the fine details of small lesions and understanding the global structure of large, sprawling ones.

**FIGURE 5:**
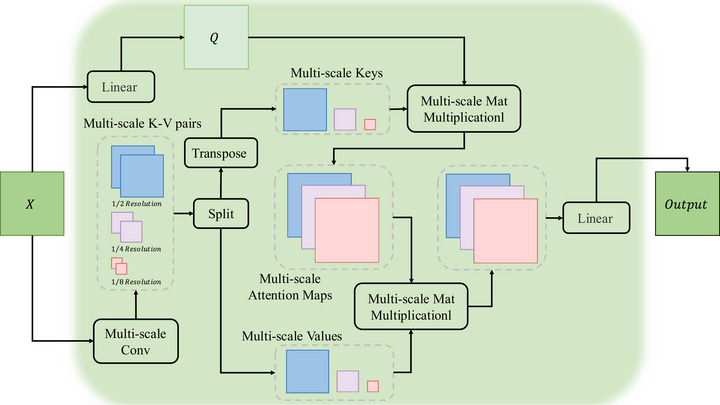
The architecture of the proposed multi‐scale query attention block.

Figure 5 illustrates the computational steps of our proposed MSQFormer module. Specifically, let the input feature map be $X\in R^{B\times C\times H\times W}$, where B is the batch size, C is the channel dimension, H is the height and W is the width. We reshape the input tensor to $X_{q}\in R^{B\times N\times C}$ first. Here, N=H×W is the number of image tokens. Then, the query matrix is generated via linear projection and reshaping:

(4) $$Q=\text{Reshape}\left({\text{Linear}}_{q}\left(X_{q}\right)\right)\in R^{B\times h\times N\times d}.$$

where, h is the number of attention heads and d=C/h is the dimension of each head. It should be noted that the query (Q) maintains the full resolution of the input feature tensor X.

Then, we generate multi‐scale key‐value matrices from the input $X\in R^{B\times C\times H\times W}$. Here, we generate spatial downsampling key‐value pairs at three (1/2, 1/4, and 1/8) different resolutions:

(5) $$F_{1}={\text{Conv2D}}_{k=2,s=2}\left(X\right)\in R^{B\times C\times H/2\times W/2}.$$

(6) $$F_{2}={\text{Conv2D}}_{k=4,s=4}\left(X\right)\in R^{B\times C\times H/4\times W/4}.$$

(7) $$F_{3}={\text{Conv2D}}_{k=8,s=8}\left(X\right)\in R^{B\times C\times H/8\times W/8}.$$

(8) $$K_{1},V_{1}=\text{Split}\left({\text{Linear}}_{kv1}\left(\text{GELU}\left(\text{LN}(F_{1})\right)\right)\right)\in R^{B\times h/2\times M_{1}\times d}.$$

(9) $$K_{2},V_{2}=\text{Split}\left({\text{Linear}}_{kv2}\left(\text{GELU}\left(\text{LN}(F_{2})\right)\right)\right)\in R^{B\times h/2\times M_{2}\times d}.$$

(10) $$K_{3},V_{3}=\text{Split}\left({\text{Linear}}_{kv3}\left(\text{GELU}\left(\text{LN}(F_{3})\right)\right)\right)\in R^{B\times h/2\times M_{3}\times d}.$$

where $M_{i}=\frac{HW}{s_{i}^{2}}$, $s_{i}$ is the stride of path i. GELU is Gaussian error linear unit activation function and LN is layer norm.

To realize local feature enhancement, each value matrix is augmented with depthwise separable convolution:

(11) $${\tilde{V}}_{i}=V_{i}+{\text{DWConv}}_{3\times 3}\left(V_{i}\right),\;i\in \left\{1,2,3\right\}$$

where ${\text{DWConv}}_{3\times 3}$ is the depthwise convolution with kernel size 3×3.

Then, we computed scaled dot‐product attention for each scale path:

(12) $$\begin{array}{cc} {\mathrm{SA}}_{i} & =\text{softmax}\left(\frac{Q_{i}K_{i}^{⊤}}{\sqrt{d}}\right)\in R^{B\times h_{i}\times N\times M_{i}},Z_{i}\\ & ={\mathrm{SA}}_{i}{\tilde{V}}_{i}\in R^{B\times h_{i}\times N\times d}. \end{array}$$

where, ${SA}_{i}$ is the self‐attention weights of the path i and $Z_{i}$ is the contextual features of path i.

Finally, we concatenate outputs from all paths and project them to realize multi‐scale feature fusion:

(13) $$Z=\text{Concat}\left(Z_{1},Z_{2},Z_{3}\right)\in R^{B\times N\times 3C/2},$$

(14) $$O=\text{Dropout}\left({\text{Linear}}_{proj}\left(Z\right)\right)\in R^{B\times N\times C}.$$

Previous Transformer models such as Swin Transformer^9^ and PVT^22^ capture multi‐scale information implicitly through downsampling in a hierarchical feature pyramid, where fusion heavily relies on the encoder's resolution hierarchy. This approach overlooks the multi‐scale nature of objects within a single attention layer, making these models sensitive to scale variations in real‐world scenarios. The root cause lies in their attention design: each layer uses tokens with fixed receptive fields and uniform granularity, preventing simultaneous perception of multiple scales.

**FIGURE 6:**
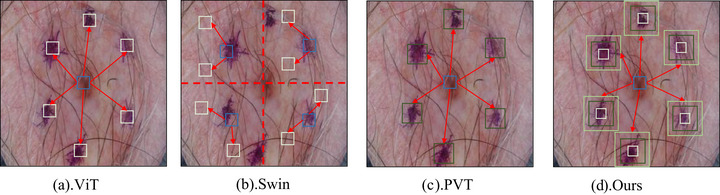
Comparison of feature modeling within a single self‐attention layer among ViT, Swin, PVT, and the proposed MSQFormer. (a)ViT): employs global self‐attention with uniform receptive fields, lacking explicit scale awareness. (b)Swin: utilizes window‐based local attention confined to fixed regions, thus limiting cross‐scale interactions within one layer. (c)PVT: encodes multi‐scale features through a hierarchical pyramid, where fusion occurs only across stages rather than inside individual attention layers. (d) Ours (MSQFormer): introduces learnable semantic queries to explicitly and adaptively aggregate information across multiple scales within a single attention layer, achieving enhanced global–local coupling.

In contrast, the proposed MSQFormer introduces explicit cross‐scale attention within each ViT block by means of learnable semantic queries. This mechanism decouples multi‐scale fusion from the encoder hierarchy, enabling content‐adaptive, sparse, and efficient aggregation of full‐scale information rather than fixed‐topology fusion across adjacent layers. Consequently, MSQFormer achieves stronger global–local coupling and greater robustness to scale variation, effectively preserving the structural integrity of large lesions while maintaining fine boundary details in small ones. As shown in Figure 6, the difference in modeling behavior across single self‐attention layers is evident: (a) ViT employs global attention with uniform receptive fields, lacking explicit scale awareness. (b) Swin restricts attention to fixed local windows, limiting cross‐scale interactions within one layer. (c) PVT encodes multi‐scale features via hierarchical downsampling, with fusion occurring only across stages. (d) Ours (MSQFormer) performs explicit, content‐adaptive cross‐scale attention using learnable semantic queries, allowing joint modeling of global context and local detail within the same layer.

### 3.4 Wavelet‐attention refinement module

Standard skip connections in U‐Net^7^ architectures directly concatenate high‐resolution, low‐level features from the encoder with semantically rich, high‐level features from the decoder. This creates a “semantic gap,” forcing the model to implicitly learn to suppress irrelevant textures and noise from the detailed encoder features. To address this challenge more explicitly, we introduce the wavelet‐attention refinement module (WARM).

The core insight behind WARM is to first structurally disentangle the low‐level features before fusing them. For this purpose, the discrete wavelet transform (DWT) is uniquely suited due to its ability to decompose a feature map into multiple sub‐bands that represent different frequency components while preserving their spatial localization. This decomposition separates coarse, low‐frequency structural information from fine‐grained, high‐frequency details (e.g., edges and textures). With features now organized into distinct sub‐bands, we can leverage the high‐level semantic context from the decoder as a precise guide. The subsequent attention mechanism can then selectively focus on the most task‐relevant frequency bands at specific spatial locations, rather than contending with a single, convoluted feature map. This guided refinement process effectively bridges the semantic gap, leading to more accurate feature fusion and superior segmentation.

**FIGURE 7:**
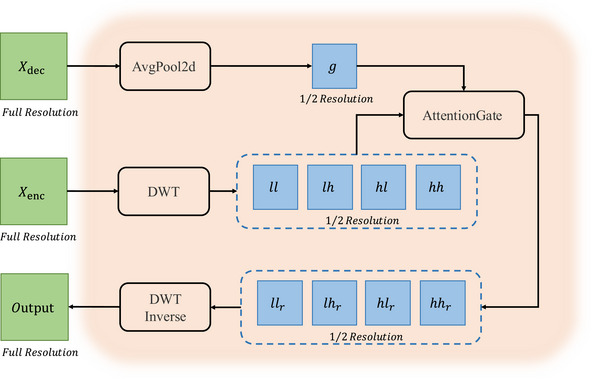
The architecture of the proposed WARM. WARM, wavelength‐attention refinement model.

As shown in Figure 7, the WARM leverages high‐level semantic information from the decoder to guide the frequency‐domain refinement of low‐level features, rather than performing a direct concatenation.

Assuming both the input low‐level feature map from the encoder, $X_{\mathrm{enc}}\in R^{B\times C\times H\times W}$, and the corresponding upsampled high‐level feature map from the decoder, $X_{\mathrm{dec}}\in R^{B\times C\times H\times W}$. The encoder feature map $X_{\mathrm{enc}}\in R^{B\times C\times H\times W}$ is passed through a DWT. This operation decomposes it into four sub‐bands, each with half the spatial resolution: one low‐frequency approximation sub‐band, ll, and three high‐frequency detail sub‐bands, lh, hl, and hh. The resulting shape for each of these four sub‐bands is $\left(B,C,\frac{H}{2},\frac{W}{2}\right)$. The above steps can be represented by formulas as:

(15) $$ll,lh,hl,hh=\text{DWT}\left(X_{\mathrm{enc}}\right)\in R^{B\times C\times \frac{H}{2}\times \frac{W}{2}}.$$

where ll,lh,hl,hh represent the low‐frequency, horizontal high‐frequency, vertical high‐frequency, and diagonal high‐frequency sub‐bands, respectively.

Concurrently, the decoder feature map $X_{\mathrm{dec}}\in R^{B\times C\times H\times W}$ is downsampled via average pooling. This process matches its spatial dimensions to those of the sub‐bands, resulting in a guidance signal $g\in R^{B\times C\times \frac{H}{2}\times \frac{W}{2}}$. The above step can be represented by formulas as:

(16) $$g=\text{AvgPool}\left(X_{\mathrm{dec}}\right)\in R^{B\times C\times \frac{H}{2}\times \frac{W}{2}}.$$

We define the attention gate function as A. The guidance signal $g\in R^{B\times C\times \frac{H}{2}\times \frac{W}{2}}$ is then used to modulate each of the four sub‐bands via separate attention gates. For an arbitrary input sub‐band $x_{\mathrm{sub}}$ and the guidance signal g, the attention gate function A can be represented by formulas as:

(17) $$A\left(x_{\mathrm{sub}},g\right)=x_{\mathrm{sub}}⊙\sigma \left(W_{\psi }\left(\text{ReLU}\left(W_{x}\left(x_{\mathrm{sub}}\right)+W_{g}\left(g\right)\right)\right)\right).$$

where $W_{x}$, $W_{g}$, and $W_{\psi }$ are the learnable weights of 1×1 convolutional layers, ReLU is the rectified linear unit activation function, σ is the sigmoid function, and ⊙ denotes element‐wise multiplication. For instance, the $ll\in R^{B\times C\times \frac{H}{2}\times \frac{W}{2}}$ sub‐band and the guidance signal $g\in R^{B\times C\times \frac{H}{2}\times \frac{W}{2}}$ are fed into the $attn_{ll}$ gate. The gate produces an attention map of shape $R^{B\times 1\times \frac{H}{2}\times \frac{W}{2}}$, which is then applied element‐wise to the ll sub‐band. This process does not change the sub‐band's shape. The output, ${ll}_{refined}$, and similarly $lh_{refined}$, $hl_{refined}$, and $hh_{refined}$, all retain the shape $R^{B\times C\times \frac{H}{2}\times \frac{W}{2}}$.

Finally, these four refined sub‐bands are recombined using an inverse discrete wavelet transform (IDWT). This transform merges the frequency components, restoring the original spatial resolution. The final output is a refined feature map, $X_{refined}$ refined, with a shape of $R^{B\times C\times H\times W}$, identical to the original input. The above step can be represented by formulas as:

(18) $$\begin{array}{cc} x_{refined} & =\text{IDWT}(ll_{refined},lh_{refined},hl_{refined},hh_{refined})\\ & \;\times \in R^{B\times C\times H\times W}. \end{array}$$

Prior boundary refinement methods^23^, ^24^ often use spatial gradients or edge maps to impose guidance losses on model outputs, which tends to confuse true boundaries with high‐frequency artifacts (e.g., hair, skin texture, illumination seams). Our WARM differs from existing methods: we decompose features in the wavelet domain into (LL/LH/HL/HH) subbands and gate them with decoding semantics, thereby enhancing only the high‐frequency subbands related to true boundaries while suppressing structured noise (such as hair and skin texture), before transforming back to the spatial domain. This subband‐specific, semantics‐guided refinement mechanism can significantly sharpen edges without amplifying pseudo‐contours and complements MSQFormer's global modeling. Through this sequence, the WARM effectively purifies the low‐level features, creating a representation that is both spatially precise and semantically informed, thereby bridging the semantic gap and significantly improve the model's segmentation accuracy at lesion boundaries.

## 4 EXPERIMENT SETTINGS

### 4.1 Dataset

To evaluate the effectiveness of our method, we conducted experiments on two publicly available benchmarks from the International skin imaging collaboration challenge: ISIC 2017, which comprises 2 150 dermoscopic images with corresponding segmentation masks, and ISIC 2018, which comprises 2 694 labeled images.

To ensure a fair comparison, we adopted the same data initialization protocol as prior works MALUNet^25^ and BDFormer.^24^ We split each dataset into 70% for training and 30% for testing. Concretely, in ISIC 2017 we used 1500 images for model training and 650 for evaluation, while in ISIC 2018 we allocated 1 886 images to the training set and 808 to the test set.

### 4.2 Evaluation metrics

Following the prior works MALUNet^25^ and BDFormer,^24^ we adopt mean intersection over union (mIoU), dice similarity score (DSC), accuracy (Acc), sensitivity (Sen), and specificity (Spe) as indicators to measure segmentation performances. The mIoU, DSC, Acc, Sen, and Spe are defined as:

(19) $$\text{mIoU}=\frac{1}{k+1}\sum_{i=0}^{k}\frac{\text{TP}}{\text{TP}+\text{FP}+\text{FN}}$$

(20) $$\text{DSC}=\frac{2\text{TP}}{2\text{TP}+\text{FP}+\text{FN}}$$

(21) $$\text{Acc}=\frac{\text{TP}+\text{TN}}{\text{TP}+\text{TN}+\text{FP}+\text{FN}}$$

(22) $$\text{Sen}=\frac{\text{TP}}{\text{TP}+\text{FN}}$$

(23) $$\text{Spe}=\frac{\text{TN}}{\text{TN}+\text{FP}}$$

where *k* is the number of target segmentation categories, TP is the true positive, TN is the true negative, FP is the false negative, and FN is the false negative pixel numbers of the result.

To further evaluate the model's computational efficiency, we employ Params, which denotes the number of model parameters, measured in millions (M), and floating point operations (GFLOPs) of the model as metrics. Both Params and GFLOPs are evaluated using an input size of 256 × 256.

### 4.3 Implement details

All experiments were conducted on a single NVIDIA GeForce RTX 2080Ti GPU. To ensure fair comparison, we adopt the data augmentation strategy from MALUNet,^25^ including vertical flipping, horizontal flipping, and random rotation. The cross‐entropy (CE) loss and dice loss are employed as the loss function. Following MALUNet,^25^ we utilize the AdamW optimizer with an initial learning rate of 0.001, coupled with a cosine annealing scheduler configured with a 300‐period cycle and minimum learning rate of 1e‐5. Models were trained for 300 epochs with a batch size of 2.

## 5 RESULTS

### 5.1 Comparison with competitive models

To validate the effectiveness of our proposed method, Tables 1, 2, 3 present comparative results between our approach and current competitive (SOTA) methods on ISIC 2017^26^ and ISIC 2018^27^ datasets, including both accuracy metrics and model complexity.

**TABLE 1:** Comparative experimental results on ISIC 2017 dataset.

Model	Year	mIoU↑	DSC↑	Acc↑	Spe↑	Sen↑
UNet^7^	2015	80.07 ± 0.24	88.38 ± 0.14	93.67 ± 0.08	98.04 ± 0.11	73.51 ± 0.19
UNet++^17^	2018	81.14 ± 0.23	89.11 ± 0.13	93.97 ± 0.08	97.81 ± 0.10	76.26 ± 0.17
SANet^28^	2021	83.57 ± 0.25	90.74 ± 0.14	94.44 ± 0.08	95.93 ± 0.11	**87.56** ± **0.18**
TransFuse^13^	2021	83.84 ± 0.24	90.91 ± 0.15	94.61 ± 0.07	96.38 ± 0.10	86.44 ± 0.16
Trans‐UNet^12^	2021	85.94 ± 0.23	92.20 ± 0.14	95.50 ± 0.08	97.66 ± 0.08	85.52 ± 0.17
MAL‐UNet^25^	2022	84.67 ± 0.24	91.41 ± 0.13	95.08 ± 0.08	97.58 ± 0.07	83.52 ± 0.18
Swin‐UNet^10^	2022	81.79 ± 0.25	89.54 ± 0.15	94.01 ± 0.08	96.90 ± 0.09	80.67 ± 0.16
DCSA‐UNet^29^	2023	84.83 ± 0.23	91.51 ± 0.12	95.15 ± 0.06	97.73 ± 0.08	83.24 ± 0.17
Xbound‐Former^23^	2023	84.55 ± 0.23	91.34 ± 0.12	95.01 ± 0.06	97.33 ± 0.09	84.23 ± 0.16
Focal‐UNETR^30^	2023	84.97 ± 0.24	91.6 ± 0.13	95.17 ± 0.07	97.58 ± 0.07	84.05 ± 0.18
MSCA‐Net^31^	2023	85.38 ± 0.22	91.86 ± 0.14	95.22 ± 0.07	97.03 ± 0.09	86.88 ± 0.16
I2U‐Net^32^	2024	85.68 ± 0.25	92.04 ± 0.15	95.47 ± 0.08	98.02 ± 0.08	83.70 ± 0.15
CSCA‐UNet^33^	2024	85.57 ± 0.26	91.98 ± 0.16	95.32 ± 0.08	97.27 ± 0.11	86.34 ± 0.18
BDFormer^24^	2025	85.94 ± 0.22	92.21 ± 0.14	95.46 ± 0.07	97.40 ± 0.12	86.51 ± 0.18
HCViT‐Net(Ours)	—	**87.76** ± **0.19**	**93.30** ± **0.12**	**96.20** ± **0.05**	**98.60** ± **0.04**	85.13 ± 0.13

*Note*: Bold is the best and underline is the second best.

**TABLE 2:** Comparative experimental results on ISIC 2018 dataset.

Model	Year	mIoU↑	DSC↑	Acc↑	Spe↑	Sen↑
UNet^7^	2015	80.72 ± 0.25	88.85 ± 0.28	93.58 ± 0.07	96.56 ± 0.12	79.82 ± 0.21
UNet++^17^	2018	82.11 ± 0.26	89.76 ± 0.31	94.27 ± 0.08	**97.73** ± **0.13**	78.31 ± 0.23
SANet^28^	2021	83.52 ± 0.27	90.73 ± 0.29	94.21 ± 0.07	97.05 ± 0.14	82.69 ± 0.27
TransFuse^13^	2021	83.20 ± 0.20	90.47 ± 0.27	94.61 ± 0.08	97.66 ± 0.16	80.53 ± 0.26
TransUNet^12^	2021	85.19 ± 0.24	91.84 ± 0.23	93.94 ± 0.09	96.52 ± 0.15	86.23 ± 0.27
MALUNet^25^	2022	84.97 ± 0.22	91.71 ± 0.26	93.85 ± 0.05	96.51 ± 0.12	85.89 ± 0.27
Swin‐UNet^10^	2022	83.11 ± 0.20	90.42 ± 0.27	94.55 ± 0.09	97.44 ± 0.16	81.17 ± 0.25
DCSAU‐Net^29^	2023	84.57 ± 0.20	91.46 ± 0.20	93.65 ± 0.06	96.28 ± 0.11	85.79 ± 0.23
Xbound‐Former^23^	2023	85.12 ± 0.23	91.80 ± 0.22	93.91 ± 0.06	96.45 ± 0.10	86.29 ± 0.21
FocalUNETR^30^	2023	84.97 ± 0.21	91.71 ± 0.20	93.82 ± 0.06	96.28 ± 0.14	86.50 ± 0.27
MSCA‐Net^31^	2023	85.24 ± 0.23	91.87 ± 0.21	93.98 ± 0.05	96.65 ± 0.12	85.99 ± 0.21
I2U‐Net^32^	2024	85.66 ± 0.22	92.13 ± 0.26	94.14 ± 0.05	96.49 ± 0.12	87.08 ± 0.27
BDFormer^24^	2025	86.28 ± 0.24	92.51 ± 0.22	94.35 ± 0.07	96.10 ± 0.13	**89.14** ± **0.22**
HCViT‐Net(Ours)	—	**87.45** ± **0.18**	**93.19** ± **0.19**	**94.95** ± **0.05**	97.26 ± 0.09	88.05 ± 0.21

*Note*: Bold is the best and underline is the second best.

**TABLE 3:** Comparison of Params, FLOPs, and Inference Time (IT) at Pi5.

Model	Year	Params↓	FLOPs↓	IT@Pi5↓
UNet^7^	2015	31.04M	48.33G	5.0s
UNet++^17^	2018	36.62M	138.37G	14.3s
SANet^28^	2021	23.90M	11.99G	1.2s
TransFuse^13^	2021	31.87M	12.65G	2.3s
TransUNet^12^	2021	105.32M	38.55G	4.8s
Swin‐UNet^10^	2022	27.17M	9.42G	1.3s
I2U‐Net^32^	2024	29.65M	8.42G	0.9s
CSCA‐UNet^32^	2024	35.28M	11.94G	1.3s
BDFormer^24^	2025	103.83M	54.31G	5.6s
HCViT‐Net(Ours)	—	**5.76M**	**7.51G**	**0.8s**

*Note*: Bold is the best and underline is the second best.

#### 5.1.1 Results on ISIC 2017 dataset

As evidenced by the comprehensive experimental results on the ISIC 2017 dataset presented in Table 1, our proposed HCViT‐Net establishes new SOTA performance across critical evaluation metrics such as mIoU, DSC, Acc, and Spe when compared to 14 prominent medical image segmentation models spanning a decade of research (2015–2025). HCViT‐Net achieves a breakthrough mIoU of 87.76%, surpassing the previous best performer BDFormer^24^ (85.94%) by a margin of 1.82%. In terms of DSC, our model attains 93.30%, exceeding BDFormer's^24^ best result of 92.21% by 1.09% improvement. It is noteworthy that Table 3 presents a comparison of complexity between our proposed model and other models. Compared with the previous SOTA method BDFormer,^24^ our model uses only 5.5% of its parameters (5.76M vs. 103.83M) and 13.8% of its computational cost (7.51G vs. 54.31G). The exceptional results highlight HCViT‐Net's significant advancements over diverse architectural paradigms. Our approach outperforms ViT‐based models such as Swin‐UNet^10^ (+5.97% mIoU) and TransFuse^13^ (+3.92% mIoU), while also exceeding attention‐enhanced CNN variants like SANet^28^ (+4.19% mIoU). Furthermore, HCViT‐Net demonstrates clear superiority over the latest innovations in the field, outperforming I2U‐Net^32^ by 2.08%. Particularly noteworthy is our model's exceptional boundary segmentation capability, evidenced by the 3.21% mIoU advantage over the recent boundary‐focused method Xbound‐Former.^23^

#### 5.1.2 Results on ISIC 2018 dataset

As demonstrated by the extensive comparative analysis in Table 2, our proposed HCViT‐Net establishes new SOTA performance on the ISIC 2018 dataset, surpassing 16 leading medical image segmentation models spanning 2015–2025. The model achieves a breakthrough mIoU of 87.45%, improving upon the previous best performer BDFormer^24^ (86.28%) by a significant margin of 1.17%. In terms of DSC, HCViT‐Net reaches 93.19%, exceeding BDFormer's^24^ 92.51% by 0.68%. While BDFormer^24^ achieves the highest sensitivity (89.14%), HCViT‐Net delivers a competitive second‐best sensitivity of 88.05% while maintaining strong specificity (97.26%)—demonstrating superior clinical balance compared to models like UNet++^17^ which achieved 97.73% specificity but only 78.31% sensitivity. The results highlight HCViT‐Net's dominance across diverse architectural paradigms. Our approach shows remarkable gains over transformer‐based models, exceeding Swin‐UNet^10^ by 4.34% in mIoU and TransFuse^13^ by 4.25%. It also outperforms CNN innovations SANet^28^ (+3.93%). Against recent competitive methods, HCViT‐Net surpasses 2024's I2U‐Net^32^ by 1.79% in mIoU, while achieving a 2.33% advantage over boundary‐focused Xbound‐Former.^23^ These results establish HCViT‐Net as the new reference architecture for skin lesion segmentation, achieving unprecedented harmony between localization precision, diagnostic reliability, and clinical safety through its advanced feature learning capabilities.

#### 5.1.3 Comparison of Params, FLOPs, and Inference Time

As shown in Table 3, compared to other SOTA methods, our HCViT‐Net demonstrates significant advantages in terms of both model parameters, computational complexity and inference tTime (Deployed on Raspberry Pi5 with an input size of 256 × 256). HCViT‐Net achieves a remarkably low parameter count of 5.76M (bold, best), which is the lowest among all listed methods. This represents a 75.9% reduction compared to the previous most parameter‐efficient model SANet^28^ (underlined second best at 15.90M parameters). In terms of computational complexity, HCViT‐Net also sets a new benchmark with FLOPs of only 7.51G (bold, best). Compared to the previous most efficient model I2U‐Net^32^ (underlined second best at 8.42G FLOPs), our model reduces computational complexity by 10.8%. These results confirm that HCViT‐Net achieves unprecedented efficiency in lightweight design, delivering SOTA performance while requiring substantially fewer computational resources —making it exceptionally suitable for resource‐constrained devices and real‐time applications. In Table 4, we present a statistical analysis of the parameter counts and computational complexity (FLOPs) of each component in the model. As shown, the Encoder stage accounts for 79.89% of the total parameters (4.63M), with the MSFormer component contributing 73.43% (4.26M). This is primarily attributed to the high‐dimensional feature representations in the 128‐channel layers of the Encoder. In contrast, the Decoder stage comprises only 1.12M parameters (19.34%), reflecting the asymmetry between feature extraction and reconstruction in the encoder–decoder architecture. Although the WARM module has the fewest parameters (0.04M, 0.76%), it plays a crucial role in feature refinement and edge enhancement. In terms of FLOPs distribution, the computational load is relatively balanced between the Encoder and Decoder, accounting for 50.83% (3.82G) and 48.97% (3.68G), respectively. Notably, the MSFormer component contributes 49.21% and 47.58% of the computation within the Encoder and Decoder, respectively, confirming the central role of the multi‐head self‐attention mechanism in the model's computational core. The WARM module incurs minimal computational cost (0.03G, 0.34%), indicating that the wavelet‐based attention refinement mechanism effectively enhances feature quality while maintaining computational efficiency. This distribution of parameters and computation aligns with the characteristics of medical image segmentation tasks—rich semantic feature extraction through the Encoder, precise pixel‐level reconstruction in the Decoder, and improved feature fusion quality from the WARM module without significantly increasing computational overhead.

**TABLE 4:** Parameter count and FLOPs analysis of HCViT‐Net components.

Stage	Parameters(M)	Param ratio(%)	FLOPs(G)	FLOP ratio(%)
Encoder	4.63	79.89	3.82	50.83
CNN	0.11	1.95	0.05	0.67
MSFormer	4.26	73.43	3.70	49.21
Decoder	1.12	19.34	3.68	48.97
CNN	0.07	1.14	0.10	1.39
MSFormer	1.06	18.20	3.58	47.58
WARM	0.04	0.76	0.03	0.34
Complete model	5.76	100.00	7.51	100.00

### 5.2 Ablation studies

#### 5.2.1 The effect of each component in HCViT‐Net

Table 5 presents a systematic ablation study evaluating the contribution of each component in HCViT‐Net. On the ISIC 2017 dataset, the complete model achieves competitive performance (87.76% mIoU, 93.30% DSC, 96.20% Acc). Removal of the MSQFormer module causes the most significant degradation, reducing mIoU by 4.08%–83.68% and DSC by 2.54%, underscoring its fundamental role in feature extraction. Eliminating the CNN block results in a 1.86% mIoU reduction, indicating the importance of local feature modeling. The absence of the WARM module preserves relatively strong performance (86.63% mIoU). For ISIC 2018, similar trends emerge: The intact HCViT‐Net maintains optimal metrics (87.45% mIoU, 93.19% DSC, 94.95% Acc), while MSQFormer removal again causes the most severe performance drop (4.18% mIoU reduction). Notably, eliminating the CNN block disproportionately affects accuracy (0.68% decrease to 94.27%), whereas WARM ablation primarily impacts DSC (0.57% reduction). These findings demonstrate that while MSQFormer blocks are essential for high performance, the WARM module's comprehensive stage‐wise integration is crucial for maximizing segmentation accuracy.

**TABLE 5:** Ablation study of the effect of each component in HCViT‐Net.

Dataset	Method	mIoU↑	DSC↑	Acc↑
ISIC 2017	HCViT‐Net(Ours)	**87.76** ± **0.19**	**93.30** ± **0.22**	**96.20** ± **0.05**
	w/o MSQFormer	83.68 ± 0.21	90.76 ± 0.23	94.92 ± 0.06
	w/o CNN	85.90 ± 0.20	92.70 ± 0.22	95.52 ± 0.05
	w/o WARM	86.63 ± 0.21	92.92 ± 0.22	95.79 ± 0.05
ISIC 2018	HCViT‐Net(Ours)	**87.45** ± **0.18**	**93.19** ± **0.19**	**94.95** ± **0.05**
	w/o MSQFormer	83.27 ± 0.19	90.55 ± 0.23	94.27 ± 0.06
	w/o CNN	86.26 ± 0.20	92.51 ± 0.22	94.27 ± 0.06
	w/o WARM	86.47 ± 0.22	92.62 ± 0.23	94.44 ± 0.07

Abbreviation: w/o, without.

**FIGURE 8:**
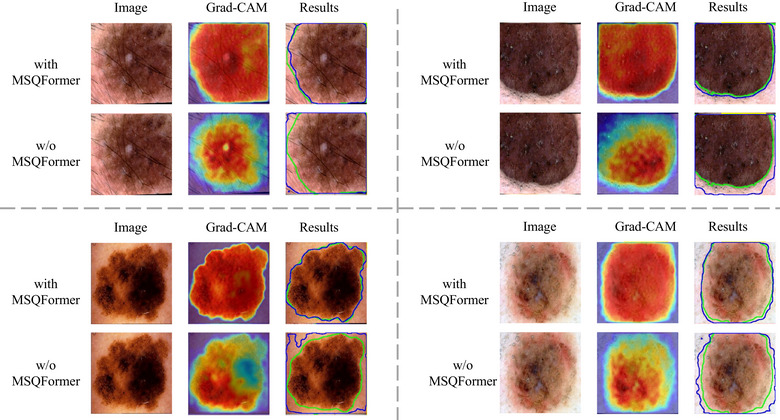
Qualitative comparison of our full model (with MSQFormer) and its ablation variant (w/o MSQFormer) on four challenging cases. From left to right, the columns display the original dermoscopy image, the Grad‐CAM attention heatmap, and the final segmentation results. In the ”Results” column, the green contour represents the ground truth, while the blue contour indicates the model's prediction. MSQFormer, multi‐scale query transformer.

In Figure 8, we verified the contribution of MSQFormer to modeling the global context through interpretable visualizations. Concretely, we removed MSQFormer from the model (w/o MSQFormer), compared it with the full model that includes this module (with MSQFormer) under identical training and evaluation protocols, and applied Grad‐CAM to the final segmentation predictions to generate heatmaps. The accompanying figure presents several representative examples. From the visualizations, adding MSQFormer yields continuous and well‐covered high responses across the entire lesion region in the Grad‐CAM maps, attending to both the center and the periphery. In contrast, removing the module leads to fragmented and locally biased activations that are often attracted to high‐contrast textures while neglecting low‐contrast or boundary areas. Regarding boundaries, the model with MSQFormer is more stable at fuzzy or irregular edges, and its predicted contours align more closely with the annotations; conversely, without the module, the predictions tend to bleed into background or miss concave parts, and the boundary responses in the heatmaps become more discontinuous. This difference is especially pronounced for lesions with strong appearance heterogeneity: with MSQFormer, the model treats the lesion as a coherent whole, whereas without it, attention concentrates on the most salient subregions, resulting in incomplete masks. Mechanistically, MSQFormer introduces long‐range dependencies explicitly via multi‐scale query aggregation, aligning coarse‐scale semantics with fine‐scale boundary cues and re‐weighting encoder features in a query‐guided manner to suppress background clutter and unify lesion responses. This matches the Grad‐CAM evidence of more uniform, coherent lesion activations and clearer, context‐consistent boundaries. Consistent quantitative results further corroborate these findings: under the same settings, incorporating MSQFormer yields improvements in mIoU of approximately 4.08% on ISIC 2017 and 4.18% on ISIC 2018. In summary, the Grad‐CAM comparisons clearly demonstrate that MSQFormer enables the model to effectively capture and exploit global context, thereby improving segmentation quality.

**FIGURE 9:**
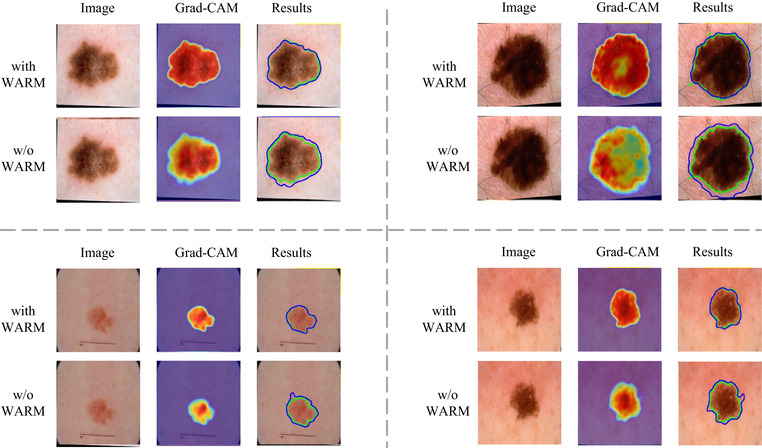
Qualitative comparison of our full model (with WARM) and its ablation variant (w/o WARM) on four challenging cases. From left to right, the columns display the original dermoscopy image, the Grad‐CAM attention heatmap, and the final segmentation results. In the ”Results” column, the green contour represents the ground truth, while the blue contour indicates the model's prediction. WARM, wavelength‐guided attention refinement module.

In Figure 9, we illustrate how the proposed WARM enhances lesion boundary precision by comparing the full model (with WARM) against an ablated variant (w/o WARM) under identical training and evaluation conditions, using Grad‐CAM heatmaps for visualization. As shown, the model with WARM produces heatmaps with tighter, more continuous, and higher‐intensity response bands that closely follow the true lesion contours, accurately capturing concave and irregular boundary structures. In contrast, removing WARM results in diffuse and unstable activations that often spill beyond the lesion edge or become over‐smoothed, leading to boundary dilation, fragmentation, or omission, especially along low‐contrast regions. Moreover, WARM effectively suppresses high‐frequency background interference—such as hair, vascular textures, and illumination variations—keeping attention concentrated around genuine lesion boundaries. The w/o WARM model, however, tends to misfocus on these pseudo‐edges, producing boundary expansion or spurious protrusions.

The benefit is particularly evident in challenging cases with low boundary contrast or strong internal heterogeneity: WARM yields more coherent and stable edge responses across the lesion, resulting in segmentation masks that align more precisely with ground truth, whereas the ablated variant exhibits fragmented activations and weakened edge energy. Mechanistically, WARM decomposes encoder features into frequency subbands—low‐frequency structural (LL) and high‐frequency detail (LH/HL/HH) components—via wavelet transform, and selectively enhances high‐frequency boundary cues using decoder‐guided gating. It then reconstructs spatial features through an inverse wavelet transform, recovering sharper and more continuous lesion boundaries. This behavior aligns with the Grad‐CAM observations of “ring‐like activations following true contours, reduced background misactivations, and tighter predicted edges.” We further employed the HD95 metric to quantitatively evaluate the improvement in boundary refinement brought by the WARM module. As shown in Table 6, adding WARM yields an HD95 improvement of 1.22 on ISIC 2017 and 1.01 on ISIC 2018, confirming that WARM substantially enhances boundary focus and robustness, leading to more accurate and stable segmentation performance. WARM, wavelet‐attention refinement module; ISIC, International skin imaging collaboration.

**TABLE 6:** Quantitative comparison of lesion boundary segmentation accuracy (HD95) with WARM.

Dataset	Method	HD95↓
ISIC 2017	with WARM	**16.01** ± **1.73**
	w/o WARM	17.23 ± 1.69
ISIC 2018	with WARM	**16.27** ± **1.67**
	w/o WARM	17.28 ± 1.77

#### 5.2.2 The effect of appending position of MSQFormer block

As demonstrated in Table 7, appending MSQFormer across all stages consistently outperforms encoder‐only and decoder‐only strategies. On ISIC 2017, the full‐stage approach (87.76% mIoU) substantially exceeds encoder‐only (85.58%) and decoder‐only (86.06%) configurations by 2.16% and 1.70% mIoU, respectively. The same superiority holds for ISIC 2018: the full‐stage model (87.45% mIoU) stably outperforms both encoder‐only (85.29%) and decoder‐only (86.33%) variants with margins of 1.96% and 1.12%. As shown in Figure 11, the GradCAM^34^ heatmaps of the CNN block and MSQFormer block in the first block of HCViT‐Net reveal that the CNN, constrained by its local receptive field, struggles to focus on the entire target object during the early model stage. In contrast, the MSQFormer, leveraging its global receptive field, can precisely focus on the entire lesion area. This evidence confirms that the full‐stage integration of global information into CNNs yields optimal performance improvements, validating the efficacy of our HCViT‐Net architectural design.

**TABLE 7:** Ablation study of the effect of appending position of MSQFormer block.

Dataset	Method	mIoU↑	DSC↑	Acc↑
ISIC 2017	All Stages	**87.76** ± **0.19**	**93.30** ± **0.22**	**96.20** ± **0.05**
	No appending	83.68 ± 0.21	90.76 ± 0.23	94.92 ± 0.06
	Only Encoder	85.58 ± 0.21	91.98 ± 0.22	95.31 ± 0.05
	Only Decoder	86.06 ± 0.23	92.27 ± 0.23	95.59 ± 0.06
ISIC 2018	All Stages	**87.45** ± **0.18**	**93.19** ± **0.19**	**94.95** ± **0.05**
	No appending	83.27 ± 0.19	90.55 ± 0.23	94.27 ± 0.06
	Only Encoder	85.29 ± 0.21	91.91 ± 0.23	93.97 ± 0.07
	Only Decoder	86.33 ± 0.22	92.53 ± 0.24	94.44 ± 0.07

#### 5.2.3 The efficacy of multi‐scale query mechanisms in MSQFormer block

**FIGURE 10:**
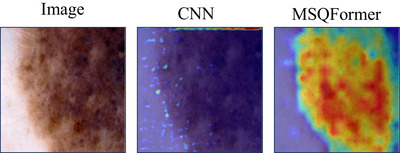
The heatmaps of the CNN block and MSQFormer block in the first stage of HCViT‐Net.

**FIGURE 11:**
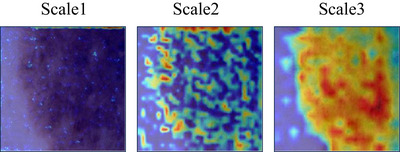
The heatmaps of multi‐scale queries within the MSQFormer block.

As shown in Table 8, on ISIC 2017 dataset, models with multi‐scale queries achieved 87.47% mIoU, 93.13% DSC, and 96.01% Acc – outperforming single‐scale baselines by 0.90%, 0.48%, and 0.08%, respectively. This advantage intensified on ISIC 2018, where multi‐scale configuration delivered gains of 0.81% mIoU, 0.47% DSC, and 0.52% Acc. Building upon Figure 10 and 11, we further visualize the GradCAM^34^ heatmaps of multi‐scale queries within the MSQFormer block, revealing differences in the model's attention granularity across scales. This demonstrates the superior efficacy of multi‐scale queries in skin lesion segmentation.

**TABLE 8:** Ablation study of the efficacy of multi‐scale query mechanisms in MSQFormer block.

Dataset	Method	mIoU↑	DSC↑	Acc↑
ISIC 2017	Multi‐scale Query	**87.47** ± **0.19**	**93.13** ± **0.12**	**96.01** ± **0.05**
	Single‐scale Query	86.57 ± 0.21	92.65 ± 0.14	95.93 ± 0.06
ISIC 2018	Multi‐scale Query	**87.45** ± **0.18**	**93.19** ± **0.19**	**94.95** ± **0.05**
	Single‐scale Query	86.64 ± 0.21	92.72 ± 0.20	94.43 ± 0.06

### 5.3 Visualization of prediction results

**FIGURE 12:**
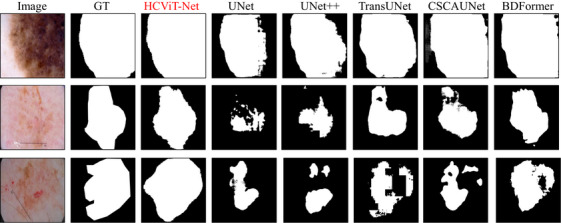
Qualitative comparisons between ours and other models on the ISIC 2017 dataset.

**FIGURE 13:**
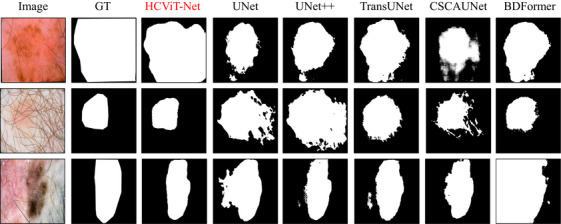
Qualitative comparisons between ours and other models on the ISIC 2018 dataset. ISIC, International skin imaging collaboration.

To further validate the effectiveness of our proposed approach, Figures 12 and 13 present a qualitative comparison of prediction results between our HCViT‐Net and current SOTA models UNet,^7^ UNet++,^17^ TransUNet,^12^ CSCAUNet,^33^ and BDFormer.^24^

The visual evidence in these two figures elaborates on these strengths: the first is our model's superior grasp of global dependencies, which significantly reduces misjudgments in large‐scale areas. Concurrently, the second is that, by synergistically combining the strengths of ViT and CNN architectures, our HCViT‐Net achieves enhanced detail processing capabilities compared to prior methods.

## 6 DISCUSSION

In this study, we introduced HCViT‐Net, a novel deep learning framework for skin lesion segmentation, and demonstrated its competitive performance on the challenging ISIC 2017 and ISIC 2018 datasets. The results confirm that our hybrid approach, which marries the local feature strengths of CNNs with the global context capabilities of Transformers, is highly effective for this complex medical imaging task.

The superior performance of HCViT‐Net stems from two key architectural innovations. The first is MSQFormer, which enables the synergistic fusion of local CNN features and global ViT context at all stages of the network. By efficiently compressing the key/value tensors, MSQFormer significantly reduces the complexity of the self‐attention mechanism, allowing its integration throughout the entire network backbone and ensuring robust representation learning from shallow to deep layers. In addition, our proposed WARM effectively bridges the semantic gap between encoder and decoder features, resulting in more precise boundary delineation and improved segmentation accuracy. This two‐stage process—robust feature fusion followed by targeted refinement—is the primary reason for the model's high accuracy, which achieved an mIoU of 87.76% on the ISIC 2017 benchmark and 87.45% on the ISIC 2018 benchmark, establishing it as a highly competitive tool for dermatological image analysis.

From a clinical perspective, the implications of an accurate and efficient segmentation tool like HCViT‐Net are significant. Automated, reliable lesion measurement provides a robust foundation for the quantitative analysis of the “ABCD” rule (asymmetry, border, color, diameter), bolstering the objectivity and confidence of clinical decision‐making. Furthermore, the model's computational efficiency is not merely a technical advantage but a critical enabler for practical adoption. Its lightweight nature makes HCViT‐Net a prime candidate for integration into standard dermatological software or even mobile health applications, where it could serve as a real‐time “second opinion” for clinicians without requiring specialized, high‐cost hardware. This could help accelerate diagnostic workflows and facilitate longitudinal monitoring of suspicious lesions.

Beyond dermoscopy, we believe the full approach (MSQFormer + WARM) is, in principle, broadly transferable, while being particularly advantageous for dermoscopic segmentation due to its close alignment with the characteristic properties of dermoscopic images. From a general applicability perspective, MSQFormer's multi‐scale queries and global modeling reliably capture long‐range dependencies and multi‐scale structures, making it suitable for medical scenarios with pronounced object size variability (e.g., polyps, glands, retinal vessels, and certain organ/tumor segmentations). Its query‐based fusion mechanism is also relatively backbone‐agnostic, facilitating integration into UNet‐like or hybrid CNN–Transformer encoders. Meanwhile, WARM employs wavelet‐domain decomposition and semantic gating to selectively enhance high‐frequency boundary cues and suppress structured noise in the frequency domain; this boundary refinement strategy is modality–agnostic in principle. Notably, in dermoscopic tasks the combination exhibits a stronger synergy: lesions often present rich, irregular, and low‐contrast boundary textures, alongside high‐frequency artifacts such as hair, skin texture, and uneven illumination. WARM strengthens true edges while suppressing spurious activations, whereas MSQFormer preserves global shape and contextual consistency amid pronounced multi‐scale variability—together forming a complementary “global–local” pairing. For modalities with smoother, plateau‐like boundary transitions (e.g., some MRI organs), the gains from WARM may be more modest; in such cases, performance can be tuned by adjusting the wavelet basis, the decomposition level J, and the gating strength. More generally, we anticipate positive yet task‐dependent gains across other medical segmentation problems, which can be further improved through lightweight adaptations, including wavelet‐level selection, gating calibration, and 3D extensions.

Nevertheless, this study has several limitations that must be acknowledged. First, the training and validation were performed exclusively on dermoscopic images, which are acquired under controlled lighting and magnification. The model's performance on standard clinical photographs, which exhibit greater variability, has not yet been assessed. To enhance generalizability for real‐world primary care settings, future work should incorporate these more diverse image types. These limitations define clear avenues for our future research, which will also include extending the framework to perform simultaneous lesion segmentation and classification.

## 7 CONCLUSIONS

In this paper, we presented HCViT‐Net, a novel hybrid architecture designed to address the core challenges of skin lesion segmentation. Its primary innovation, the MSQFormer, enables the synergistic fusion of local CNN features and global ViT context at all network stages. By efficiently compressing key/value tensors, MSQFormer facilitates powerful self‐attention with significantly reduced complexity, allowing its integration throughout the entire network backbone. This ensures robust representation learning from shallow to deep layers. Furthermore, our WARM effectively mitigates the semantic gap between encoder and decoder features, leading to more refined boundary delineation and enhanced segmentation accuracy. Experimental results on the public ISIC 2017 and ISIC 2018 benchmark datasets confirm that HCViT‐Net achieves competitive segmentation accuracy, providing a more reliable foundation for subsequent automated analysis of diagnostic features like asymmetry and border irregularity. Crucially, this high performance is achieved with notable computational efficiency, making HCViT‐Net a prime candidate for integration into clinical workflows. It holds the potential to serve as a real‐time, reliable “second opinion” for dermatologists, helping to accelerate diagnostic procedures without the need for specialized, high‐cost hardware.

## AUTHOR CONTRIBUTIONS

**Wei Jiao**: Conceptualization; methodology; software; formal analysis; investigation; data curation; writing original draft preparation; writing review and editing; visualization. **Jianghui Xu**: Conceptualization; methodology; formal analysis; writing review and editing. **Yijiao Fang**: Methodology; Formal analysis; resources; writing review and editing. **Jiaojiao Huang**: Software; data curation. **Yujie Zhu**: Writing—review and editing. **Dandan Ling**: Conceptualization; supervision; writing—review and editing. All authors have read and agreed to the published version of the manuscript.

## CONFLICT OF INTEREST STATEMENT

The authors declare no conflicts of interest.

## Data Availability

The code of our paper is available at: https://github.com/lddFDU/HCViT‐Net.
